# Photovoltaic Effect in the MnZn_1−x_Cr_x_Sb Weyl Semimetals

**DOI:** 10.3390/ma19173670

**Published:** 2026-08-28

**Authors:** Sergey Aplesnin, Oksana. Romanova, Maxim Sitnikov, Anton Kharkov, Oleg Nikitinsky, Natalia Cheremnykh, Lubov Udod, Alexander Shabanov, Grigory Rymski, Olga Minchukova, Abdelbaki Hichem

**Affiliations:** 1 Kirensky Institute of Physics, Federal Research Center Krasnoyarsk Science Center of the Siberian Branch of the Russian Academy of Sciences, Krasnoyarsk 660036, Russia; apl@iph.krasn.ru (S.A.); rob@iph.krasn.ru (O.R.); alexch_syb@mail.ru (A.S.); 2 Institute of Space Technology, Reshetnev Siberian State University of Science and Technology, Krasnoyarsk 660037, Russia; kineru@mail.ru (M.S.); khark.anton@mail.ru (A.K.); onikitinsky@yandex.ru (O.N.); natasha13082002@gmail.com (N.C.); 3 Scientific-Practical Materials Research Center NAS, 220072 Minsk, Belarus; grigorij.rimskij@mail.ru (G.R.); orion_minsk@tut.by (O.M.); 4 Research Center in Semiconductor Technology for Energetic, 02, Bd. Dr. Frantz FANON, B.P. 140, Merveilles-7, Algiers 16038, Algeria; abdel.hichem@outlook.fr

**Keywords:** weyl semimetals, photocurrent, photovoltage, photoresistance, curie temperature

## Abstract

**Highlights:**

- A correlation of the magnetization, photovoltage, and photocurrent was found
- The photocurrent increases nonlinearly with increasing light intensity
- Maximum of the photovoltaic effect is detected in the mid-IR range
- Photocurrent and photovoltage regulation by magnetic field
- Magnetophotoresistance changes sign when the polarity of the magnetic field is switched

**Abstract:**

The photovoltaic effect in the MnZn1−xCrxSb Weyl semimetals was studied in a wide spectral range. A correlation in the temperature behavior of the magnetization, photo-voltage, and photocurrent has been found and a sharp increase in the photocurrent below 140 K has been established. Increasing by 50% of the generation of photocurrent is accompanied grows of the Curie temperature by 3%. It is shown that, under the same sample illuminance, the photocurrent and photovoltage attain their maximum values under illumination with an incandescent lamp, rather than with a laser with a wavelength of 830 nm or a led lamp. The photocurrent and photovoltage are increased nonlinearly with increasing power of incident light and the maximum sensitivity is detected in the mid-IR range, increasing from 1.5 mA/W to 20 mA/W with the chromium doping concentration. As the chromium content in the MnZn1−xCrxSb semimetals grows, the drop in resistance changes to a rise under illumination. The possibility of magnetic field control of the photocurrent, photovoltage, and photoresistance with a maximum value at room temperature is demonstrated.

## 1. Introduction

Homogeneous compounds without an inversion center exhibit the bulk photovoltaic effect in zero external electric field. The efficiency of this effect can be increased by narrowing the band gap and reducing the dimensionality of a material [1,2,3]. In particular, the photocurrents detected in narrow-gap semiconductors, e.g., 1D WS_2_ nanotubes and Weyl semimetals (WSMs) with broken inversion symmetry [4], exceed those in wide-gap ferroelectric insulators by orders of magnitude. The bulk photovoltaic effect arises from the inversion asymmetry-induced transition of electrons upon absorption of photons and is related to the topological quantities with the Berry phase and Berry curvature. This fact has stimulated numerous theoretical and experimental studies aimed at finding topological WSMs [5,6,7]. The main focus has been on the theoretical examination of Weyl nodes with a gap in the electron excitation spectrum [7].

The symmetry analysis and conductivity calculation near a Weyl node with inclined cones showed a leading low-frequency power-law divergence inversely proportional to the frequency [8]. For the WSMs with the antiferromagnetic order (MnGeO_3_) and with the ferromagnetic one (PrGeAl), giant peaks for a number of non-vanishing elements of the photoconductivity tensors at photon energies below 0.2 eV were calculated [8].

Moreover, the low-frequency bulk photovoltaic effect can be tuned by doping (adding carriers) and changing the magnetization orientation. In the (Ta, Nb)(P, As) family of compounds belonging to topological WSMs, TaAs exhibits the greatest separation between nodes of different chiralities [9,10] and the photovoltaic effect in the photon energy range of about 25–350 meV significantly intensifies at longer wavelengths, which is consistent with the presence of Weyl nodes at lower energies and agrees well with the previous model calculations. The photocurrent at a wavelength of 750 nm is 0.01 mA/V [2].

The giant photocurrent was found experimentally in the type-II WSM TaIrTe_4_ at four wavelengths: 633 nm, 1.5 μm, 4 μm, and 10.6 μm [11]. The photocurrent depends linearly on the illumination power with a sensitivity of 22.4 μA∙W^−1^ in the high-power region, while at a wavelength of 4 µm, it is strikingly different in the low-power region: more than three orders of magnitude greater, reaching a maximum slope of 130.2 mA∙W^−1^. The theoretical investigations showed a qualitative difference in the low-frequency dependence of the photocurrent for type-I WSMs, which decreases proportionally to the frequency, and for type-II WSMs, in which the current diverges in the low-frequency limit ω → 0. The current generation is significantly contributed by Weyl nodes. The theoretical calculations at a wavelength of 4 μm, after separating the photocurrent response from all optical transitions, identified the contribution of the transition from the lower to upper subband on the Weyl cone (from the band 0 → 1), which accounts for more than 97% of the photocurrent response [11].

The bulk photovoltaic effect (BPV) in WSMs has attracted considerable interest from researchers due to the generation of photocurrents in the IR and THz ranges [11,12]. The current generation induced by the transition of electrons between subbands is related to the contribution of the Weyl node singularity. Strengthening the singularity will lead to an increase in the photocurrent. The Berry curvature enhances the giant photoresponse and is important for application in high-sensitivity photodetectors, especially those operating in the mid-IR and THz ranges. The photovoltage and photocurrent depend on magnetization, which can be used in passive electromagnetic field detectors.

The bulk photoelectric effect occurs in semiconductors with nonequivalent electronic states, for example, in the monolayer of the MnPSe_3_ 2D antiferromagnet, which is symmetric with respect to the spatial inversion and time reversal operations [13]. Specular reflection limits some bulk photovoltage components tensor, but the local symmetry in valleys allows finite components of the BPV photocurrent. This induces photoconductivity with the valley polarization attaining ∼300 μA/V^2^, as was observed experimentally in the MnPSe_3_ monolayer [14]. By changing the AFM vector, one can change the direction of the photovoltaic current. The current value is directly proportional to the spin–orbit coupling constant.

Topological semimetals (TSMs) with degenerate bands exhibit a number of unique properties, which depend on the type of degeneracy (WSMs with isolated Weyl points [15] and Dirac semimetals with a line of nodes forming rings in the first Brillouin zone [16,17]). Topological materials are characterized by low energy consumption during electrical conduction [18,19,20], since they are resistant to carrier scattering by impurities. Magnetic TSMs can be used in spintronics due to the combination of topology, quantum transport, and magnetism. The TSM states are formed by a combination of time-reversal symmetry and non-symmorphic two-fold rotational axis symmetry, which maintains the in-plane nodal surface dependent on the magnetization direction [21].

In the Cu_2_Sb-type layered Mn_1−x_Zn_x_Sb magnetic intermetallic compounds [22,23,24,25], the electron excitation spectrum was calculated by the projection augmented-wave method [26] with the generalized gradient approximation (GGA) [27] and the Perdew–Burke–Ernzerhof (PBE) functions. The spin-polarized branches of spin-up and spin-down electron excitations and the nodal points of intersection of these spectrum branches were found, which form the nodal lines and topological properties. The spin–orbit coupling will lift the degeneracy in the region of intersection of the spin–polarized branches, leading to a shift of nodes in the first Brillouin zone. According to the results of the ab initio calculation [26], the nodes are formed by the intersection of d_xy_ and dz^2^ hybridized orbitals. Substitution of chromium for zinc ions will change the electron spectrum, since the dz^2^ orbital of the chromium ion is unoccupied and the spin–orbit coupling constant is different.

Photoelectric effects have not been investigated in the family of Mn_2_Sb magnetic intermetallics. Data on the photoelectric characteristics of Weyl semimetals at temperatures above room temperature are currently lacking. From this perspective, the study of MnZn_1−x_Cr_x_Sb semimetals is a promising line of research.

The main objective of this study was to establish the direct current generation under the action of electromagnetic radiation in a wide frequency range and the correlation of the photovoltaic effect with the magnetic symmetry in the MnZn_1−x_Cr_x_Sb solid solutions. Since the generated current depends on the magnetic characteristics, number of Weyl nodes, and their energies relative to the Fermi level, the magnetization and the Fermi level can be changed by chemical substitution of 3d elements.

## 2. Materials and Methods

### 2.1. Sample Preparation of the MnZn_1−x_Cr_x_Sb Solid Solutions

The MnZn_1−x_Cr_x_Sb solid solutions were synthesized on the basis of the MnZnSb compound by the solid-state reaction in evacuated quartz ampoules at a temperature of 973 K. The synthesized samples were subjected to thermobaric treatment at a pressure of ~5.0 GPa and a temperature of ~623 K for 120 s. The synthesis method was described in detail in [28].

### 2.2. X-Ray Diffraction and Morphology of the MnZn_1−x_Cr_x_Sb Solid Solutions

**Figure 1:**
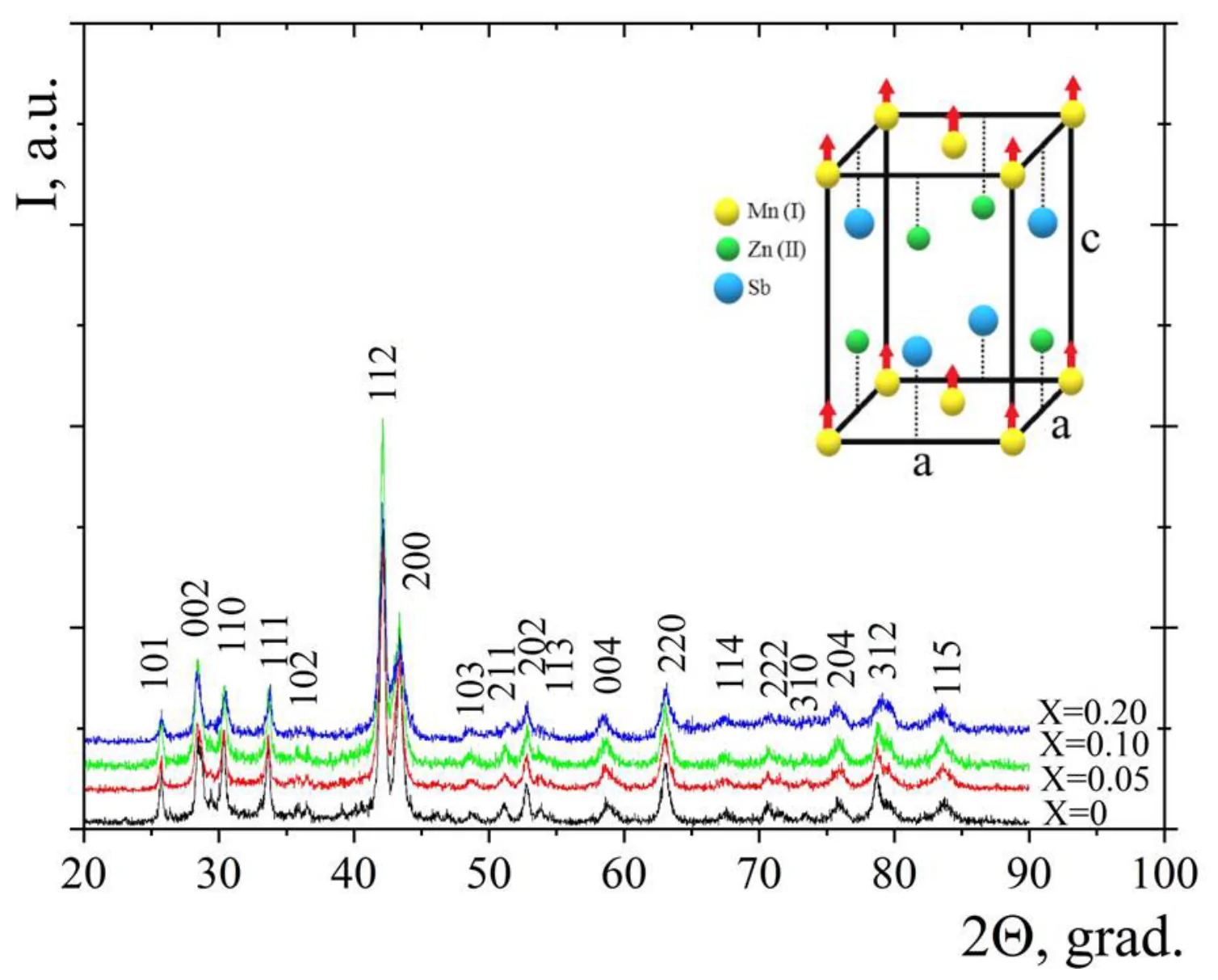
X-ray diffraction data for solid solutions of the MnZn_1−x_Cr_x_Sb system. Insert: Crystal structure of MnZnSb.

X-ray diffraction (XRD) analysis was performed on a DRON-3M diffractometer (CuKα radiation) at room temperature. The structural parameters were calculated using the FullProf program. According to the X-ray structural analysis data, the MnZn_1−x_Cr_x_Sb (0 < x < 0.1) solid solutions after the thermobaric treatment are single-phase with a Cu_2_Sb-type (anti-PbFCl) tetragonal crystallographic structure, sp. gr. P4/nmm (Figure 1), similar to the initial MnZnSb compound, the unit cell of which is shown in the insert of Figure 1. An increase in the concentration of substitution of chromium atoms for zinc ones slightly monotonically decreases the lattice parameters *a* and *c*: from *a* = 0.418 nm and *c* = 0.627 nm at *x* = 0 to *a* = 0.415 nm and *c* = 0.625 nm at *x* = 0.2. This is most likely due to the difference in atomic radii of the substitution elements. The Zn atomic radius is ~1.33 Å and the Cr atomic radius is slightly smaller, ~1.28 Å. In the Cu_2_Sb-type (P4/nmm) structure, atoms are arranged in layers [29]. The substitution of chromium for zinc occurs at a specific crystallographic site within this layered structure. When a smaller Cr atom replaces a larger Zn atom, local contraction occurs in the lattice. The parameter *a* is determined by bonds in the basal plane (for example, in the Zn–Sb or Mn–Sb layers). The parameter *c* determines the spacing between these layers. The in-plane compression can lead to a slight lattice contraction along the *c* axis.

**Figure 2:**
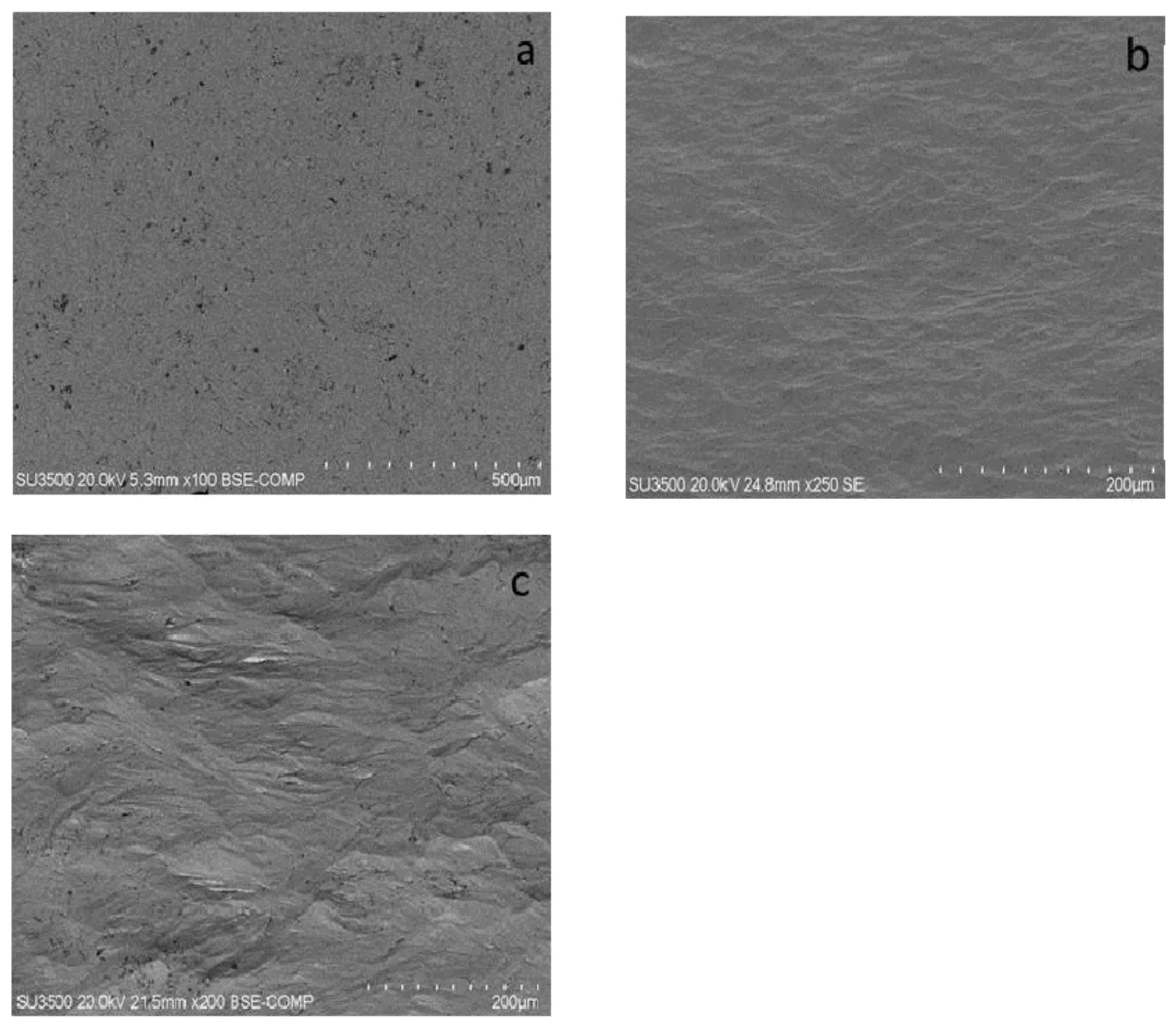
Micrographs of surface fragments of solid solutions MnZn_0.95_Cr_0.05_Sb (**a**) MnZn_0.9_Cr_0.1_Sb (**b**) and MnZn_0.8_Cr_0.2_Sb (**c**).

The chemical composition, morphology, and microstructure of the MnZn_1−x_Cr_x_Sb (0 < x < 0.1) samples were studied on a Hitachi SU-3500/Model3500 ultra-high-resolution (up to 3 nm) scanning electron microscope. Figure 2 shows the typical morphology of the samples obtained by hot pressing, possessing a high-stability powder. It can be seen that the samples have a dense, consolidated structure with a pronounced volumetric character. The surface of individual particles seems quite dense, without signs of high nanoporosity, i.e., the main structural elements of the sample are micron and submicron particles, rather than loose nanoglomerates. A key point is that the bulk morphology of the sample does not undergo significant changes under the thermobaric treatment. This indicates the mechanical stability of the MnZn_1−x_Cr_x_Sb frame, which maintains its integrity and does not deteriorate or crack during additional processing.

The results of the semi-quantitative analysis of the MnZn_1−x_Cr_x_Sb solid solutions are given in Table 1. The XRD and electron microscopy data indicate the incorporation of chromium atoms in the tetragonal crystallographic structure. With increasing chromium concentration, the manganese and zinc non-stoichiometry can arise. At x = 0.1, manganese is partially replaced by antimony and, at x = 0.2, zinc ions are replaced by manganese, which occupies the second position in the unit cell.

**Figure 3:**
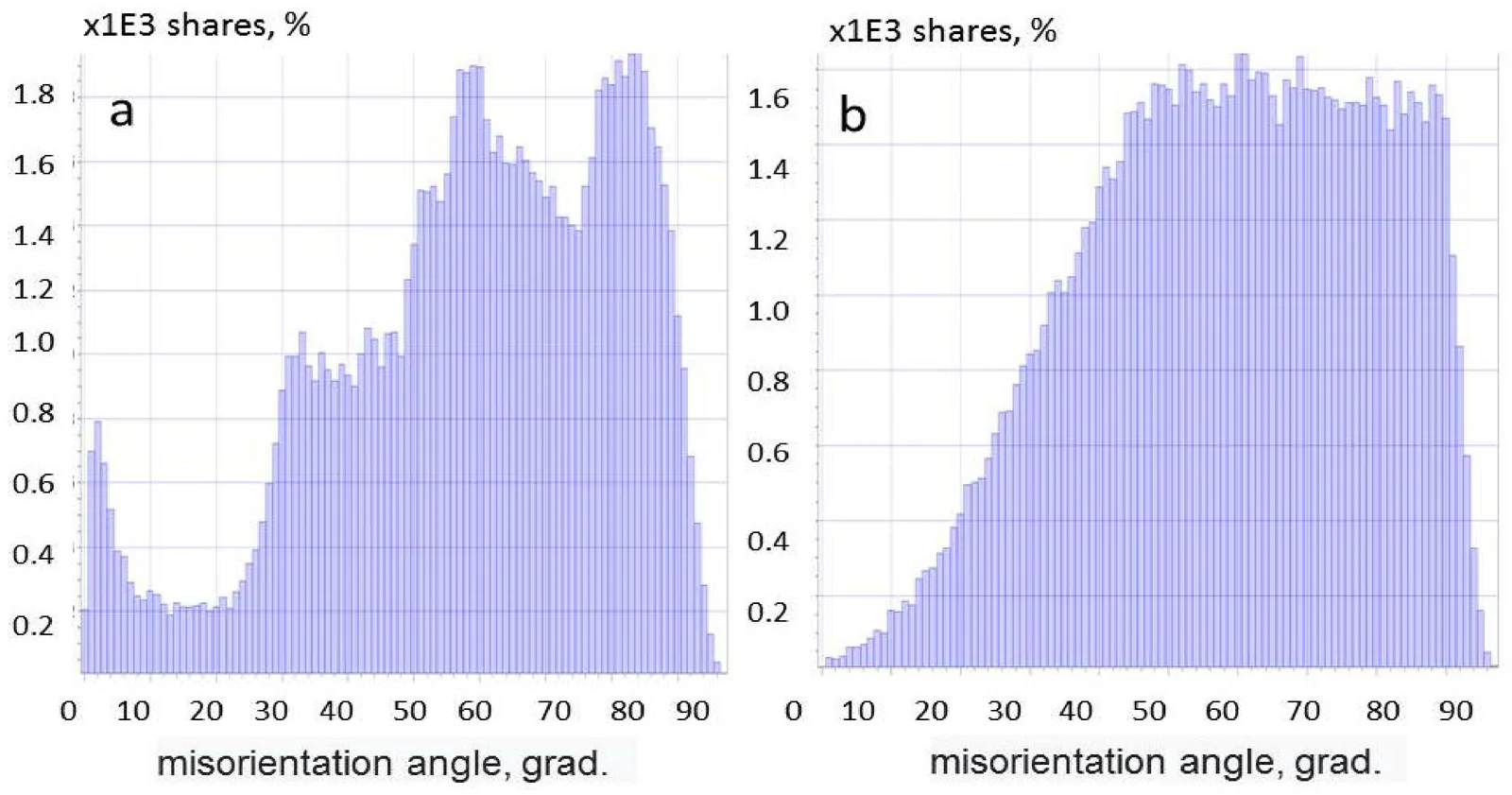
The angle of grain misorientation depending on their percentage content on the surface of the MnZn_1−x_Cr_x_Sb sample, x = 0.05 (**a**) and x = 0.2 (**b**).

Analysis of the electron microscopy data allowed us to determine the misorientation angle and thereby characterize the angle between the crystal lattices of adjacent grains. The misorientation can be two-level: low-angle (LA) and high-angle (HA) [30]. Figure 3a and Figure 3b present the angle of grain misorientation depending on their percentage content on the surface of the MnZn_1−x_Cr_x_Sb samples with x = 0.05 and x = 0.2, respectively. At a concentration of x = 0.05, several maxima are observed. The first maximum, lying in the region with small orientation angles (less than 10–15°), is caused by dislocations, which form an intergrain boundary described by the Burgess formula. They have a low energy barrier to diffusion through the sample. Two maxima in the high-angle boundaries at 60 and 85 determine the crystallographic orientation of the tetragonal axes of grains. In the latter case, the tetragonal axes of grains are approximately orthogonal. In the MnZn_0.8_Cr_0.2_Sb sample, the misorientation angle gradually increases and remains almost unchanged above 50. The high-angle boundaries represent the most effective barrier to dislocation motion. The misorientation angle significantly affects the energy of a boundary, its mobility, electronic structure, and location of Weyl nodes relative to the chemical potential, as well as the magnetic properties in weak magnetic fields.

### 2.3. Magnetization, Photocurrent, Photovoltage and Electrical Resistance

Temperature dependences of the specific magnetization of the MnZn_1−x_Cr_x_Sb (0 ≤ x ≤ 0.20) solid solutions were obtained by the ponderomotive method in a magnetic field of 8.6 kOe in the temperature range of 80–600 K. The generated photocurrent is measured using a Keithley 6517B electrometer with an accuracy of 1 nA that was manufactured by Keithley Instruments, which is in Solon, Ohio, in the United States. The voltage is measured using an Agilent 34420A instrument with a sensitivity of 0.1 nV/100 nΩ, that was manufactured by Keysight Technologies, which is in Bayan Lepas, Penang, in the Malaysia. 

The photocurrent and photovoltage were determined depending on the illumination intensity of an incandescent lamp, which has a continuous spectrum similar to the radiation of an absolutely black body. As electromagnetic radiation sources, an incandescent lamp with a power of 100 and 150 W, a near-IR laser with a wavelength of 830 nm, an LED lamp with a wavelength range of 380–780 nm, and linearly polarized light were used. In the far-IR spectral range (400–7000 cm), an IR radiation source of a Fourier spectrometer was used. The photovoltage and photocurrent were measured at fixed temperatures on the MnZn_1−x_Cr_x_Sb samples with the chromium concentration ranging within 0 < x < 0.20. The electrical voltage (current) over time was measured by periodically turning the light source on and off at intervals of one minute. After turning off the lighting, the electric voltage (current) decreased exponentially and was fitted by the function JJ0=A·exp−ττ1+B·exp−ττ2, where τ_1_ and τ_2_ are the relaxation times. The surface of the sample was illuminated by different light sources. Four electrodes were applied on the sample surface at the rectangle vertices. The sample surface was covered with thermal insulating material (mica), with a hole for illumination. The contacts were not illuminated. On one side, with two contacts, the photocurrent was measured; on the other, the photovoltage (closed circuit) was measured parallel to each other. Light falls perpendicularly onto the sample; the magnetic field is directed parallel to the current. The light source was a 150 W incandescent lamp and a 10 W LED lamp with adjustable voltage from zero to 220 V. The IR laser range at 830 nm has an input voltage from 2.0 V to 5 V with power up to 1 W. The emission spectrum of a cold-glow LED lamp is in the range of 380–780 nm with a maximum at 650 nm. The IR laser power was regulated within the range of 0.01–1 W. Illumination in the cryostat was measured using a luxmeter.

Electrical resistance from time measured using the four-probe method of Van der Pauw on the sample surface (8 × 6 × 2 mm) with periodic switching on and off of the light source. A stabilized constant current of 1 mA was used. Resistance was measured at fixed temperatures without light for two minutes, with light for four minutes. Illumination of the sample changes its electrical resistance, which is determined by us as ∆R = (R_(light)_ − R_(0)_)/R_(0)_.

## 3. Results and Discussion

### 3.1. Magnetic Properties

**Figure 4:**
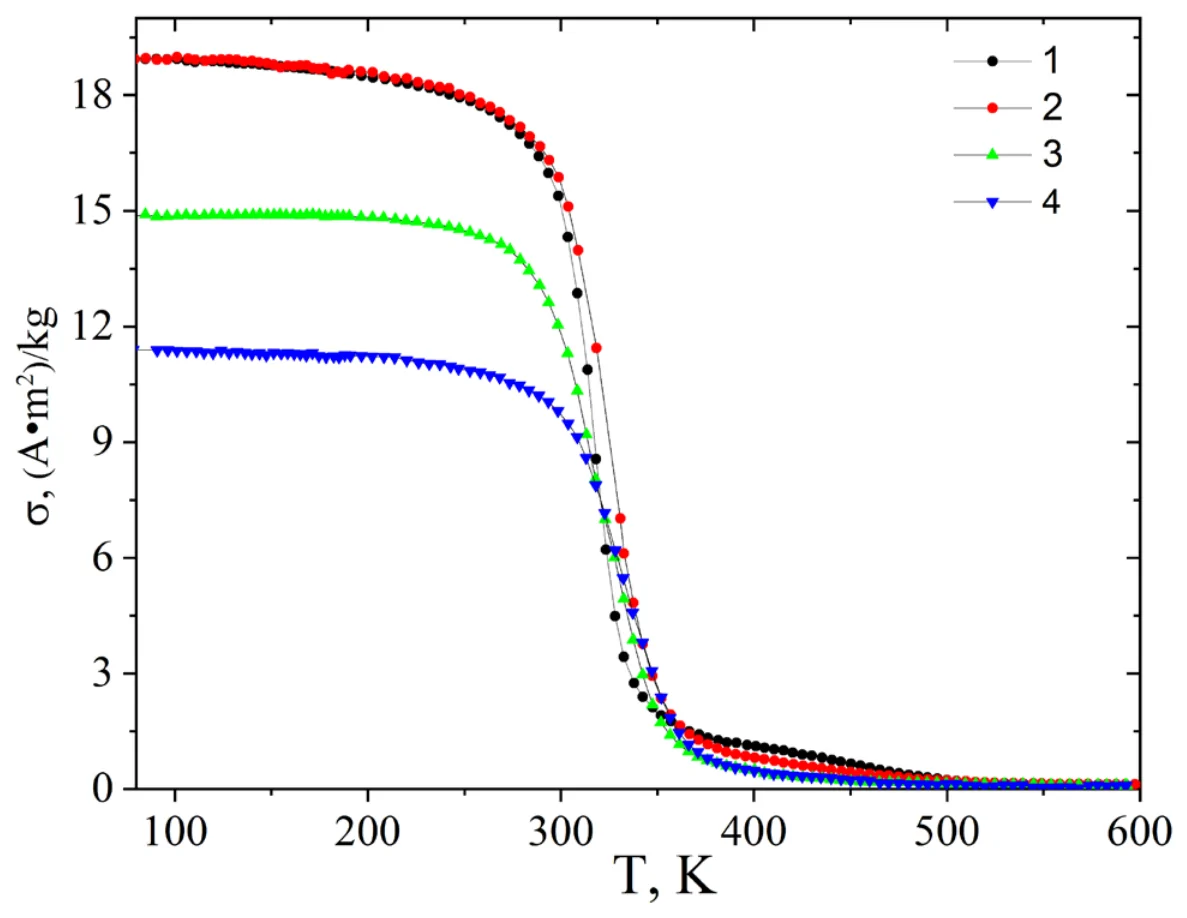
Temperature dependence of specific magnetization of MnZn_1−x_Cr_x_Sb solid solutions in a magnetic field for x = 0 (1); 0.05 (2); 0.1 (3) and 0.2 (4).

In Figure 4, one can see that the specific magnetization of the MnZn_1−x_Cr_x_Sb samples decreases with increasing chromium concentration as a result of chromium doping.

In this case, the Curie temperature T_C_ of the MnZn_1−x_Cr_x_Sb solid solutions determined by extrapolating the linear part of the temperature dependence of the squared specific magnetization to the temperature axis σ^2^ = f(T) increases from T_C_ = 323 K at x = 0 to T_C_ = 337 K at x = 0.2 with an increase in the substitution concentration. The Curie temperature 320 K of the MnZnSb compound is consistent with the data reported in [31,32]. The high magnetization value above T_C_ is possibly related to the formation of individual magnetically ordered clusters formed as a result of the nonstoichiometric distribution of elements throughout the sample volume.

### 3.2. Photovoltage and Photocurrent

**Figure 5:**
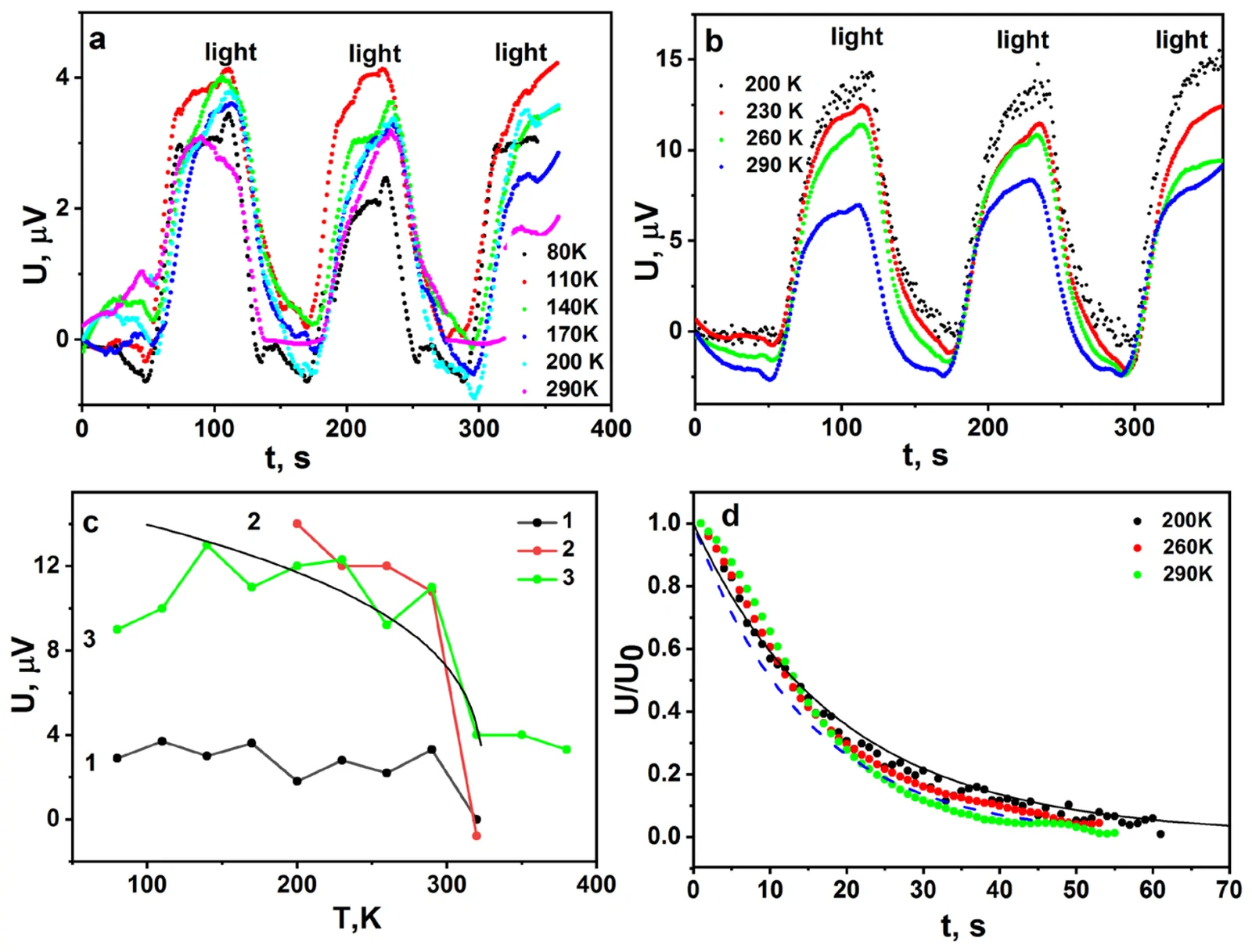
Photovoltage of the MnZn_0.95_Cr_0.05_Sb sample generated upon illumination with polarized light with a period of 60 s (**a**) and illumination with a diode lamp (**b**). Temperature dependence of the photovoltage generated upon illumination with polarized light (1), an LED lamp (2) and a laser (3) (**c**). Photovoltage after turning off the illumination as a function of time (**d**). Fitting functions Ut=U01·exp−ττ1+U02·exp−ττ2 at different temperatures T = 200 K (black), T = 290 K (blue).

Figure 5 shows the time dependences of the photovoltage obtained under periodic switching of the light sources on and off. Good repeatability of the generated voltage under switching on and off is observed for the MnZn_0.95_Cr_0.05_Sb sample (Figure 5a). The time dependence of the photovoltage is approximated by two relaxation times: Ut=U01·exp−ττ1+U02·exp−ττ2, which satisfactorily describes the experiment at τ_1_ = 15 s and τ_2_ = 25 s (Figure 5d). The two relaxation times are associated with two channels of electron and hole recombination during photoexcitation. Near the surface of the sample, where the captured charge carriers (holes or electrons) remain for a significant time, the recombination mechanisms are slow. Two types of traps with different energies determine two relaxation times and their temperature dependence in the Shockley–Read–Hall model [33].

Also, the two relaxation times are associated with the presence of four pairs of Weyl nodes that exist in the vicinity of the Fermi level with energies of ±0.04 eV and ±0.09 eV. The relaxation time τ_1_ is associated with the recombination of electrons and holes between a pair of Weyl nodes with energies of ±0.04 eV and τ_2_ with ±0.09 eV [26,34]. There is no theoretical study of relaxation time that accounts for inhomogeneous magnetic and electronic structures, domain structure, vacancies, and defects—factors that generate local built-in fields and spatially separate electrons and holes. Idealized models fail to describe the experimental results.

The corresponding relaxation times were observed when studying the photocurrent in the BiFeO_3_ films, which depend on the light wavelength. In particular, under irradiation with green light, the relaxation time is ~15 s and, with red light, ~60 s [35]. When the MnZn_0.95_Cr_0.05_Sb sample is heated to 290 K, the relaxation times decrease to ~12 s and ~22 s, respectively.

When the sample is illuminated with plane-polarized light, the relaxation time is shorter as compared with the case of illumination with a polarizer-free LED lamp. The photovoltage is independent of the polarizer plane’s orientation relative to the magnetization. A decrease in the photovoltage when illuminated with the polarized light is caused by a twofold decrease in the polarized light intensity and by light absorption in the polarizer. When illuminated with a laser, the photovoltage has a maximum at 140 K. Regardless of the light wavelength and polarization, the photovoltage decreases sharply near the Curie temperature (Figure 5c). In the proximity of the Curie temperature, the photovoltage disappears under low illumination and drops sharply under laser illumination.

**Figure 6:**
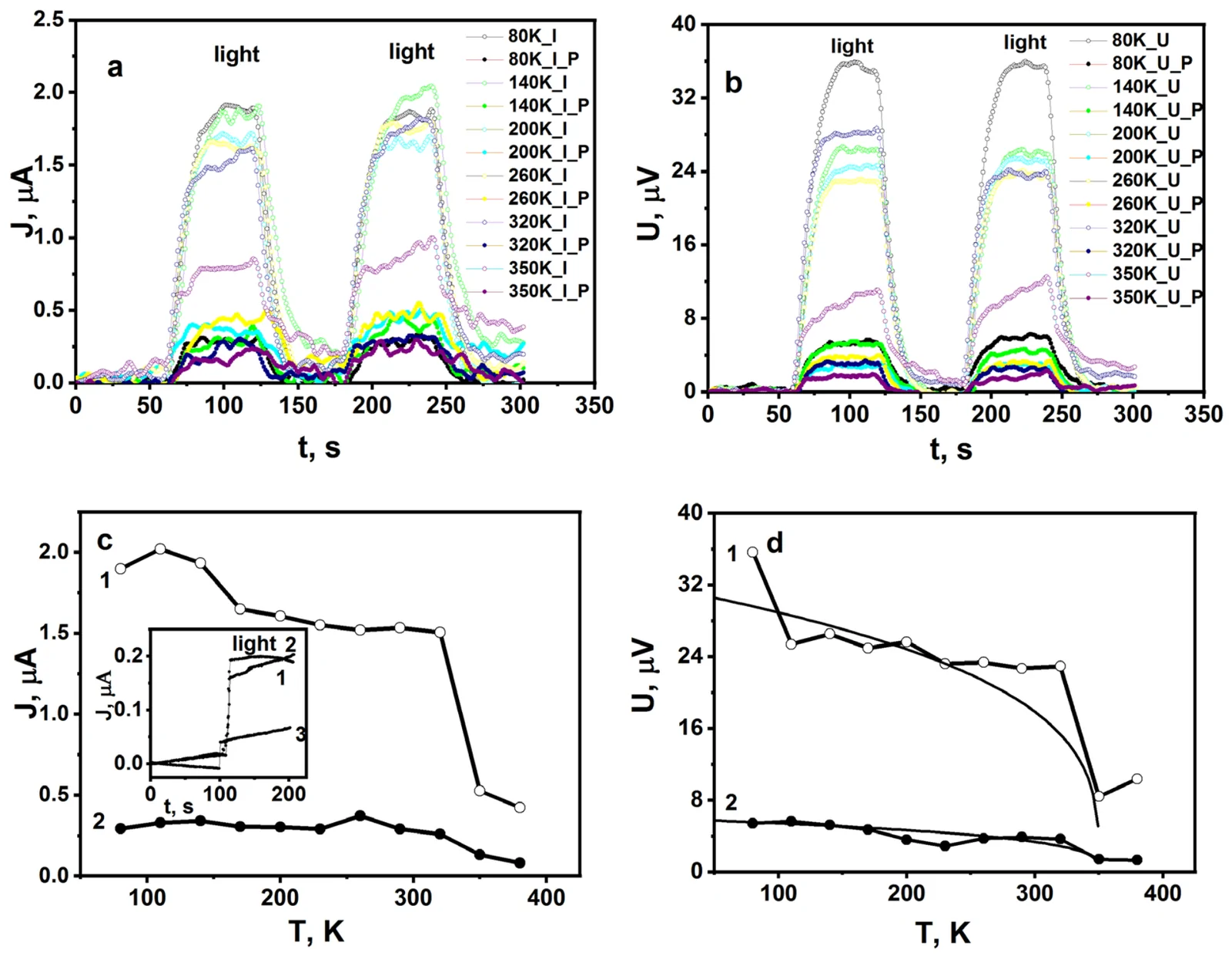
Photocurrent (**a**,**c**) and the photovoltage (**b**,**d**) of the MnZn_0.9_Cr_0.1_Sb sample as a function of time and temperature when illuminated by an incandescent lamp with a power of 48 mW/cm^2^ without a polarizer (1, I) and with a polarizer (2, P). Insert: photocurrent as a function of time when illuminated in the IR range (2–4) μm. Curve 1 corresponds to T = 110 K, 2—140 K, 3—170 K.

The use of an incandescent lamp as a light source increases the sample’s irradiation spectral range. Figure 6 shows the current and voltage of the MnZn_0.9_Cr_0.1_Sb sample induced by light over a wide wavelength range. When the light is turned on, the current and voltage increase exponentially and also relax when the light is turned off. This sample has two relaxation times at low temperatures: τ_1_ ~15 s and τ_2_ ~25 s. Upon heating, these times gradually decrease to τ_1_ ~7 s and τ_2_ ~16 s at T = 320 K and reach values of τ_1_ ~4 s and τ_2_ ~11 s at 350 K. The bulk photocurrent at zero external voltage indicates that light-induced carriers move mainly in one direction, i.e., the sample exhibits the photoelectric effect.

Narrowing of the illumination spectral range in the IR region (500–7000 cm^−1^) leads to a sharp current jump when the lighting is turned on (Figure 6). Since the IR intensity of the IR spectrometer is low, a weak current is induced. At low temperatures, the current decreases at ~160 K and the photovoltage decreases at 110 K. Further heating to the Curie temperature leads to a sharp decrease in the photocurrent and photovoltage, the temperature dependences of which are described by the power function U=U01−TTC13, as in the case of the magnetization M=M01−TTC13, i.e., the photocurrent and photovoltage are proportional to the magnetization.

**Figure 7:**
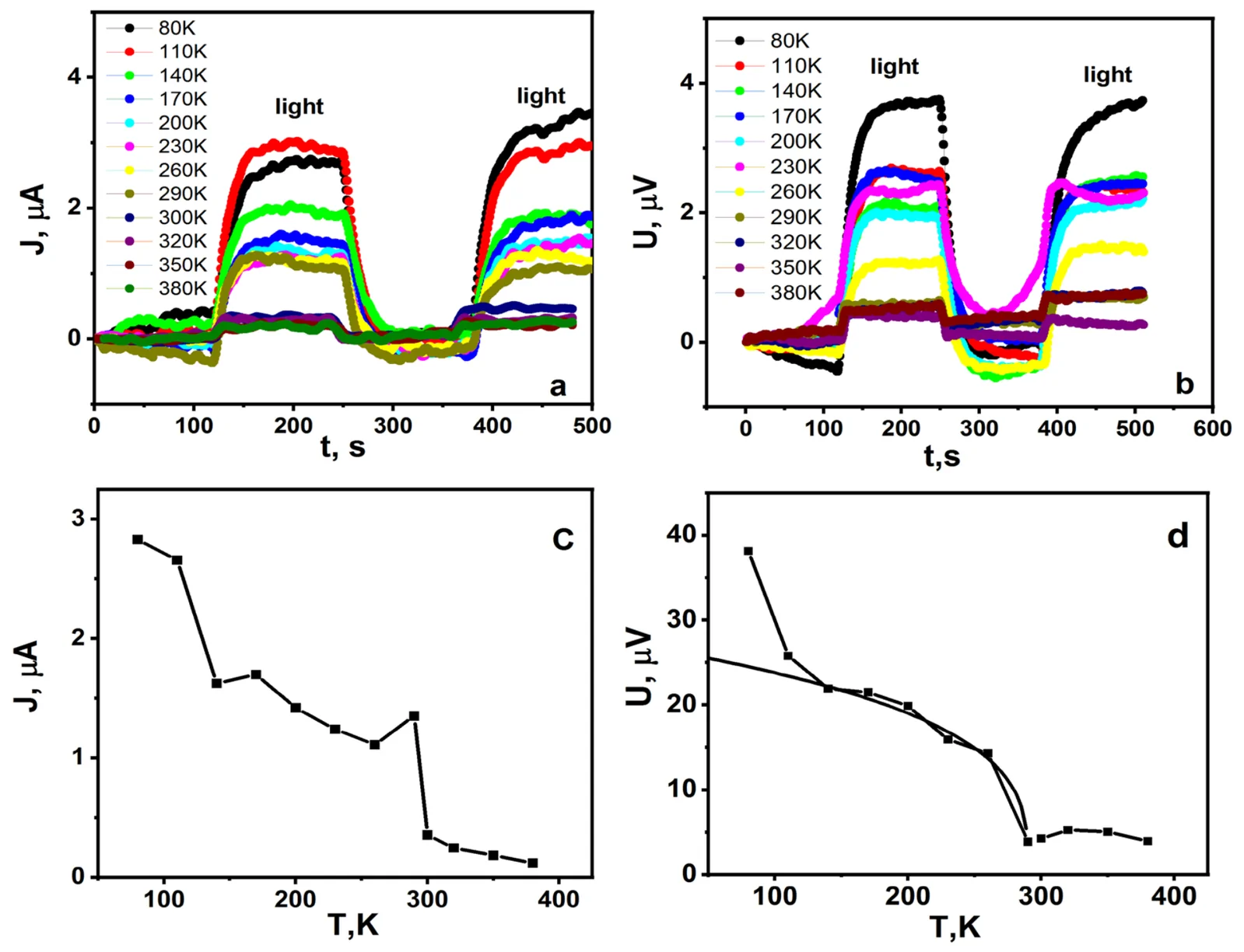
Photocurrent (**a**,**c**) and photovoltage (**b**,**d**) of the MnZn_0.85_Cr_0.15_Sb sample as a function of time and temperature when illuminated by a 60 mW/cm^2^ LED lamp.

As the chromium concentration grows, the photoelectric effect increases at low temperatures. The qualitative time dependences of the photovoltage and photocurrent (Figure 7a,b) in the MnZn_0.85_Cr_0.15_Sb samples illuminated with a led lamp are similar. During repeated switching on of the lamp, the photovoltage and current are reproduced. Here, the two relaxation times τ_1_ ~ 15 s and τ_2_ ~ 28 s are also observed, which decrease with temperature. The temperature dependences of the photovoltage and photocurrent are presented in Figure 7c,d. In the temperature range of 110–140 K, the photovoltage and photocurrent decrease by half. This is possibly due to the spin-reorientation transition at temperatures around 140 K and to a change in the spin topology [36].

**Figure 8:**
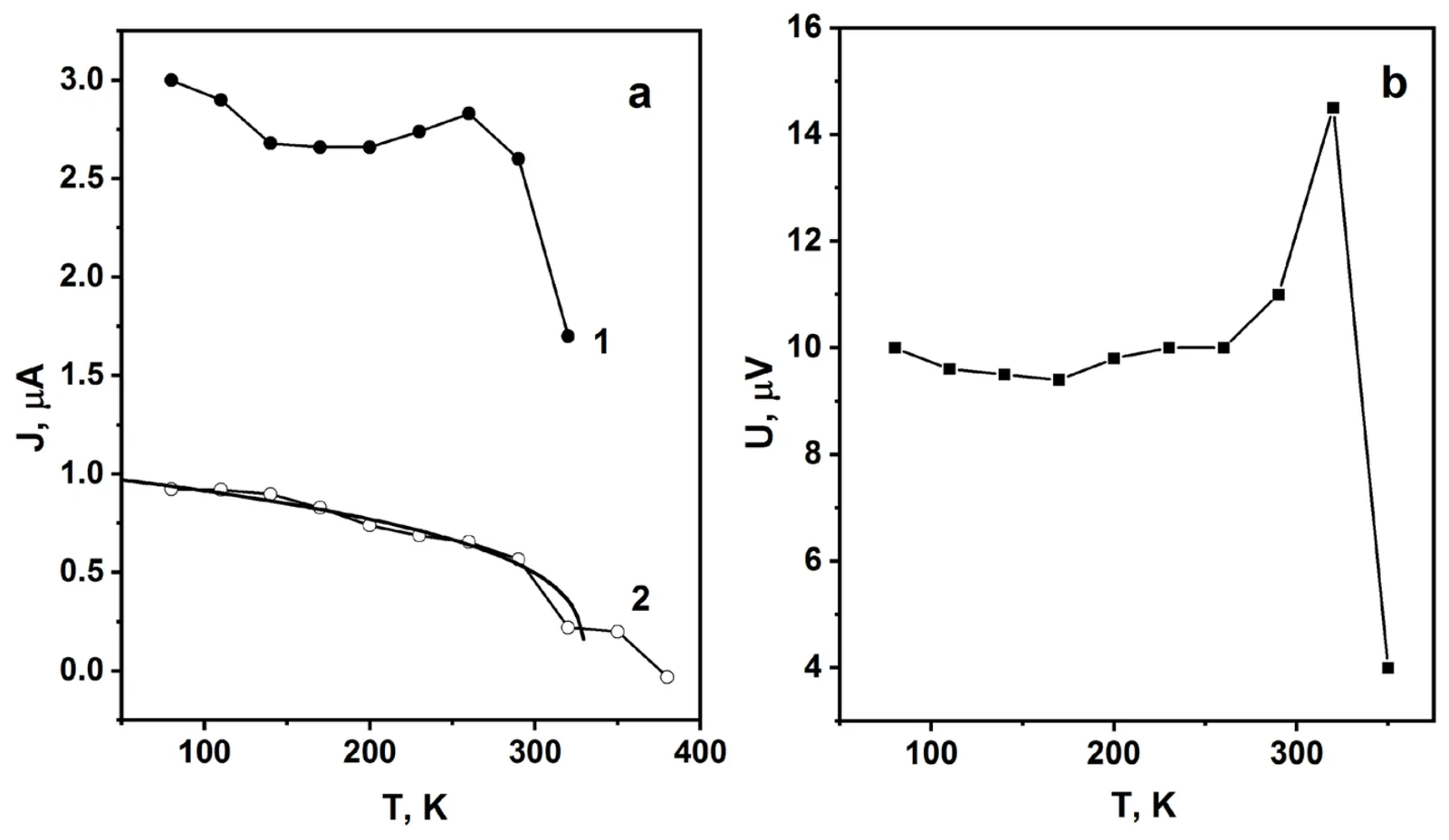
Photocurrent (**a**) and photovoltage (**b**) of the Mn_Zn0.8_Cr_0.2_Sb sample as a function of temperature under illumination with a lamp with a power of 48 mW/cm^2^ (1) and a laser with a power of 20 mW/cm^2^ (2).

Figure 8 shows the photovoltage and photocurrent in the MnZn_0.8_Cr_0.2_Sb sample. The temperature coefficient of the photocurrent has two maxima (dJ/dT 1/J) at T = 170 and 320 K (Figure 8b); above 380 K, the photocurrent vanishes. The increased photocurrent as compared with the case of laser illumination is caused by absorption of low-energy photons. The photocurrent is qualitatively described by a power-law dependence for the magnetization: M=M01−TTC13.

The photocurrent and photovoltage arise from the asymmetry of the density of electronic states near Weyl nodes relative to the center of the Brillouin zone as a result of the nonstoichiometry of the distribution of manganese, zinc, and chromium ions or vacancies and defects over the sample.

### 3.3. Effect of a Magnetic Field on the Photovoltaic Characteristics

**Figure 9:**
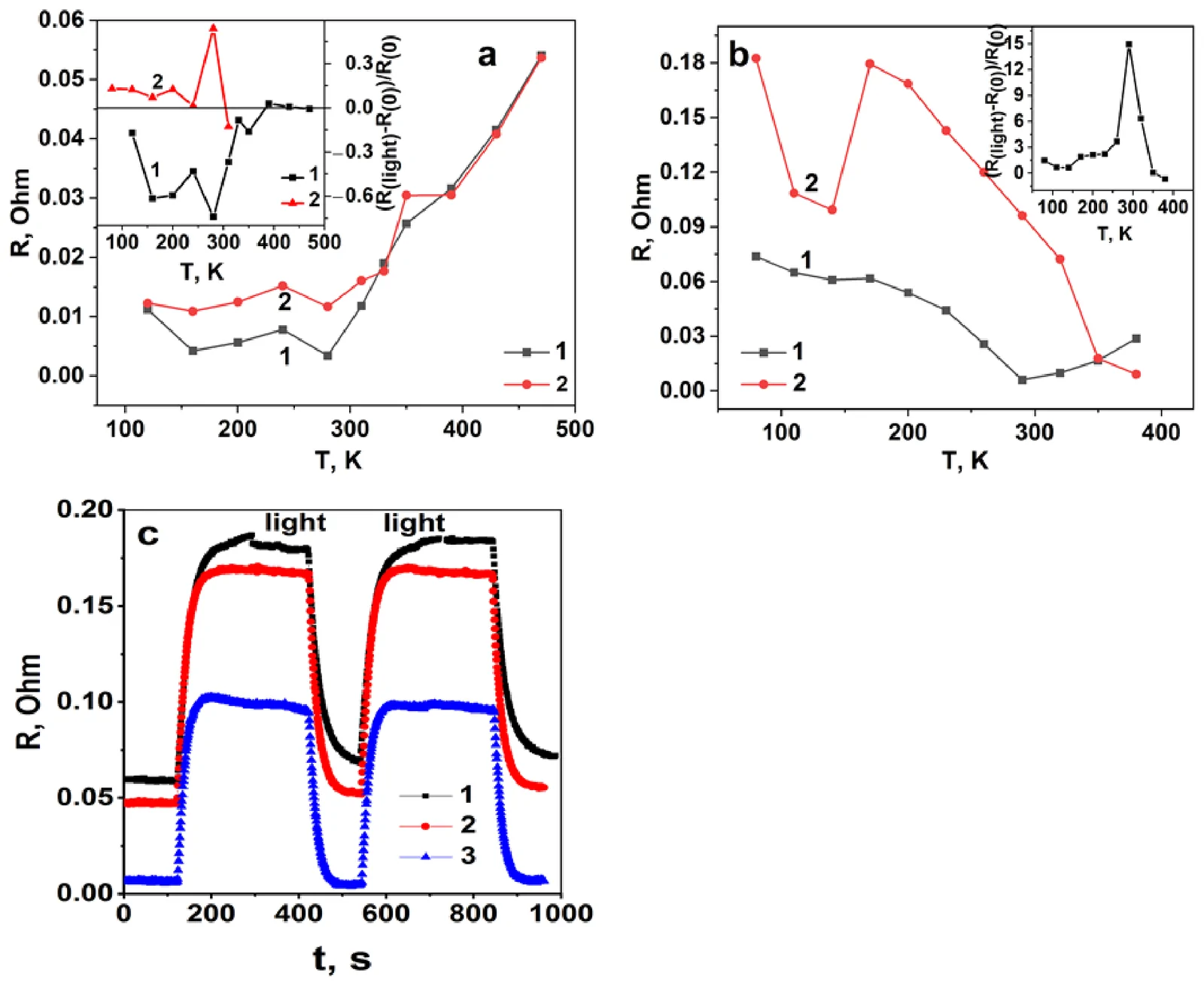
Resistance (**a**) of the MnZn_0.9_Cr_0.1_Sb, without illumination (2), under illumination (1). Insert: Photoresistance of the MnZn_0.9_Cr_0.1_Sb (1) and MnZn_0.8_Cr_0.2_Sb (2) under illumination as a function of temperature. Resistance (**b**) of the MnZn_0.85_Cr_0.15_Sb, without illumination (1), under illumination (2). Insert: Photoresistance of the MnZn_0.85_Cr_0.15_Sb. Resistance (**c**) of the MnZn_0.85_Cr_0.15_Sb when turning on and off the light at T = 80 K (1), 200 K (2), 290 K (3).

The resistance of the MnZn_1−x_Cr_x_Sb semimetals decreases at x = 0.1 and increases with increasing chromium concentration (x > 0.1, see Figure 9) under illumination by the plane-polarized light. The photoresistance ∆R (T) has extrema in the low-temperature range of 140–170 K and at 290 K. In the MnZnSb samples, a breaking crystal of symmetry at low temperatures and a spin-orientation transition near 140 K were assumed [36,37,38,39]. The feature at 290 K can be related to a topological transition, which was observed in the Mn_2−x_Zn_x_Sb solid solution at temperatures of up to 250 K [38].

The great topological Hall effect can be related to the formation of a skyrmion lattice [40,41,42]. At a chromium concentration of x = 0.1, the Fermi level is located near Weyl nodes. Linearly polarized light can be represented as two circularly polarized beams with the right- and left-hand polarization. In this case, the photocurrent arises that is perpendicular to the incident light direction, with allowance for the spin-dependent terms linear and quadratic or cubic with respect to the quasi-momentum in the electron effective Hamiltonian [43,44].

An increase in the chromium concentration causes a shift of the Fermi level away from Weyl nodes and the bias current induced by interband electronic transitions dominates. In the electron excitation spectrum, the conical dispersion can be interpreted as valleys. Electrons near different valleys experience reflection in opposite directions at the barrier edge when the energy dispersion is tilted along one of the transverse directions [45,46,47]. Strong scattering of electrons to the high-energy valleys reduces their velocity and increases the resistance [48].

**Figure 10:**
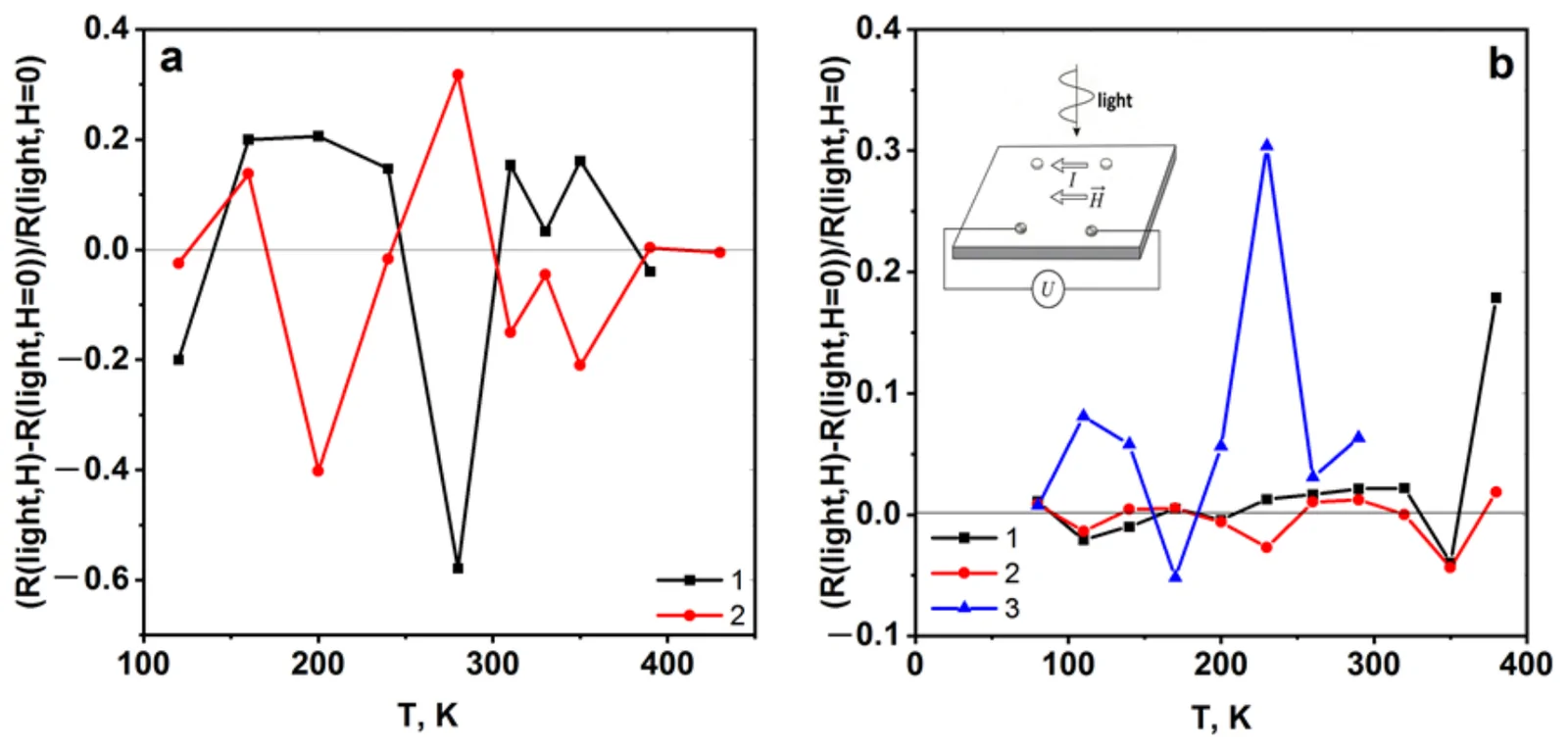
Photoresistance of MnZn_0.9_Cr_0.1_Sb in the magnetic field H = 1.2 kOe (1) and H = −1.2 kOe (2) (**a**), MnZn_0.85_Cr_0.15_Sb, H = 1.2 kOe (1), H = −1.2 kOe (2) and MnZn_0.8_Cr_0.2_Sb (3) (**b**). Inset: the measurement scheme of photovoltage and photocurrent.

In topological semimetals, the inherent coupling between the spin and momentum of an electron allows the polarized light to excite electrons with a specific helicity, thereby generating the effective spin-polarized and axial currents (the spin currents along the light propagation direction). The photocurrent is induced in a magnetic field. The photocurrent density is $J_{x}=\left(S_{xx}H_{x}+S_{xy}H_{y}\right)E^{2}$, where coefficients S_xx_ and S_xy_ are the even functions of magnetic field *H* [43]. Since in the experiment we had *H_y_* = 0, the direction of the magnetically induced current can be changed by changing the magnetic field polarity, and the contribution to the external current will be added or subtracted accordingly. As a result, the photoresistance change $\left(R_{\left(light,\;H\right)}-R_{\left(light,\;H=0\right)}\right)/R_{\left(light,\;H=0\right)}$ in the MnZn_0.9_Cr_0.1_Sb sample, changes its sign in a magnetic field when the direction of the latter changes (Figure 10a). The magnetic field is directed parallel to the current. The schematic of the experimental setup given on insert Fig.10b. For the unpolarized light and high concentrations, the change in the magnetic field direction barely affects the photocurrent magnetoresistance (Figure 10b). The resistance of the MnZn_0.8_Cr_0.2_Sb sample increases under illumination in a magnetic field.

The magnetoresistance growth is caused by the presence of the nodal annular dispersion in the vicinity of a Weyl node, which leads to closing of cyclotron orbits under exposure to a magnetic field [40,49].

**Figure 11:**
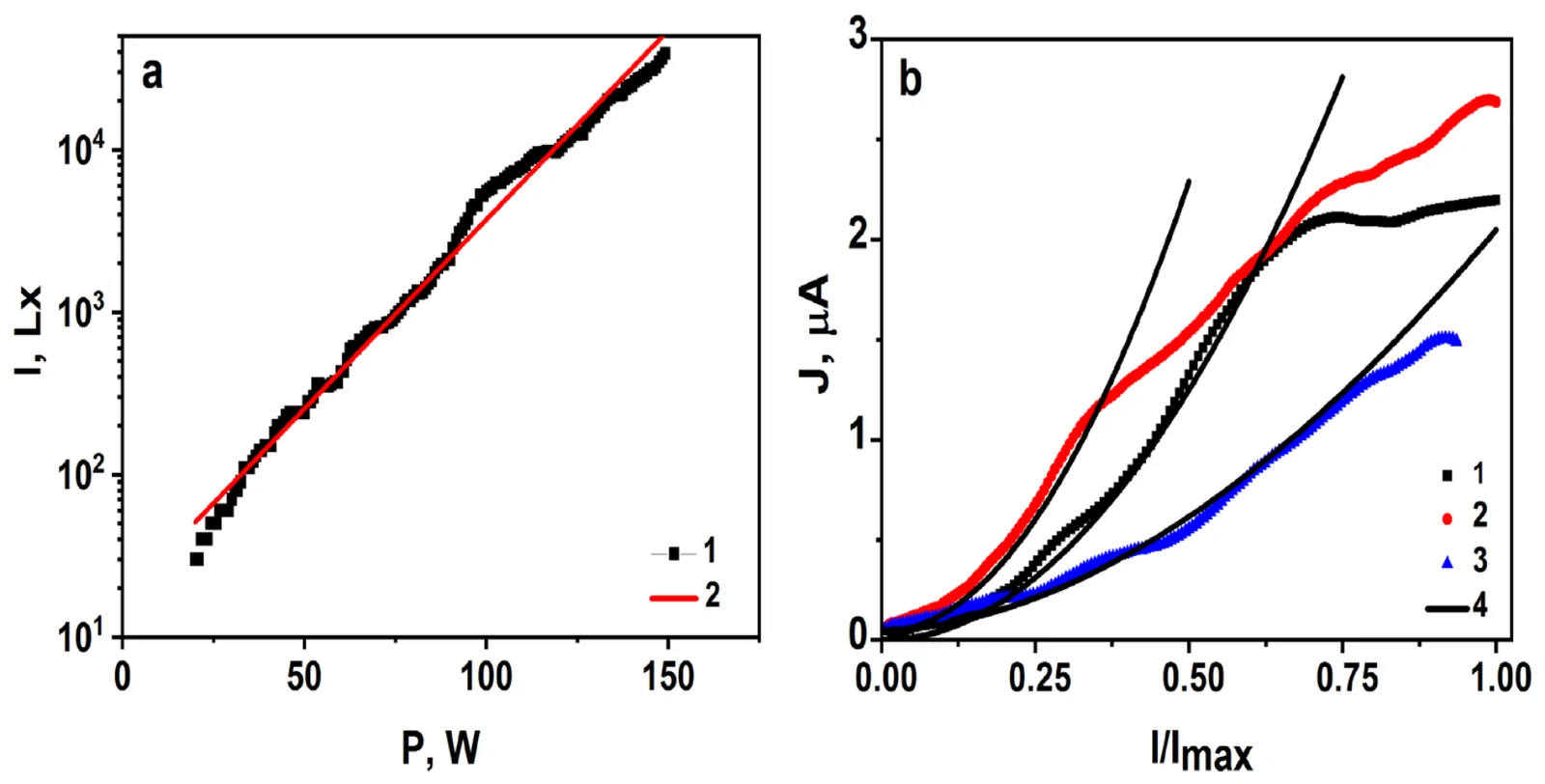
The dependence of the illumination intensity on the radiation power of an incandescent lamp of MnZn_0.9_Cr_0.1_Sb (1), approximating functions I = A exp(0.023 P) (2) (**a**). The photocurrent of MnZn_0.9_Cr_0.1_Sb determined by the normalized power of incident illumination at T = 110 K (1), 140 K (2), 170 K (3), approximating functions J = A (I/I_max_)^2^ (4) (**b**).

Weyl semimetals can be used as electrical energy sources converting electromagnetic radiation into electric energy. The emission spectrum of an incandescent electric lamp corresponds to the solar radiation spectrum. The dependences of the photocurrent and photovoltage on the lamp radiation intensity were measured in an optical cryostat. The sample illuminance was measured with a luxmeter in the wavelength range of 400–1100 nm at intensities from zero to 40,000 lux. Figure 11a illustrates the sample illuminance versus the incandescent lamp power, which is described by the exponent I=A·exp(0.023·P). The maximum power of incident light per sample is 2 mW. The sample heating by ∆T = 0.1–0.3 K is estimated from the quantity $Q=\int \left(IS\right)dt=mC_{V}∆T$, where S is the sample area and Cv is the heat capacity.

Figure 11b shows the dependence of the photocurrent on the power of incident illumination of the MnZn_0.9_Cr_0.1_Sb sample. The photocurrent is described by the quadratic dependence on the normalized power of incident illumination: $J=AI^{2}$ at T = 110 K up to 1.25 mW, T = 140 K up to 0.8 mW, and at T = 170 K up to 1.75 mW.

**Figure 12:**
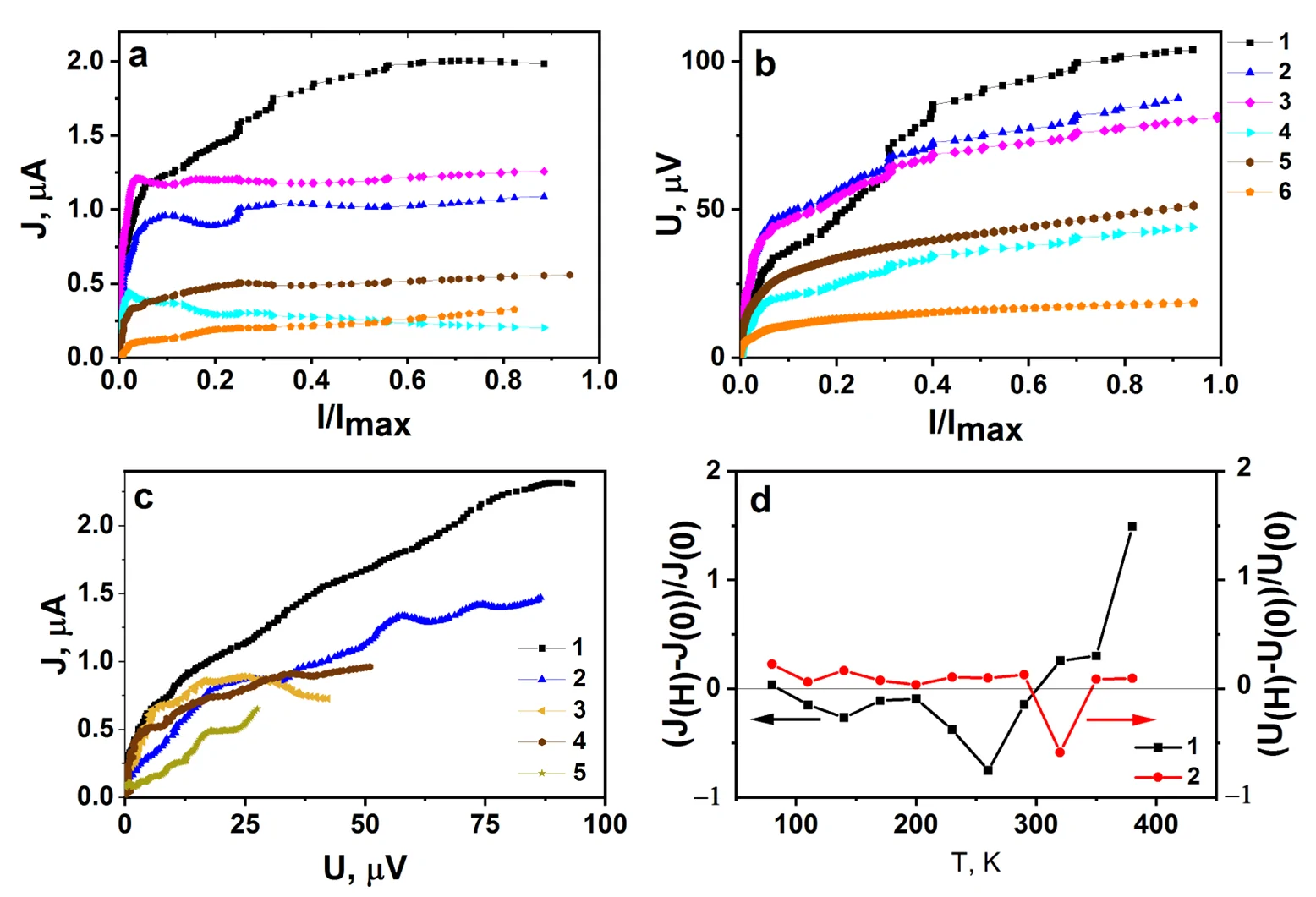
The photocurrent of the MnZn_0.85_Cr_0.15_Sb at H = 1.2 kOe, T = 80 K (1), 140 K (2), 200 K (3), 290 K (4), 320 K (5), 350 K (6) (**a**). The photovoltage on the normalized power of incident illumination of the MnZn_0.85_Cr_0.15_Sb H = 1.2 kOe at T = 80 K (1), 140 K (2), 200 K (3), 290 K (4), 320 K (5), 350 K (6) (**b**), The photocurrent versus photovoltage of the MnZn_0.85_Cr_0.15_Sb sample in a magnetic field H = 1.2 kOe at T = 80 K (1), 140 K (2), 230 K (3), 290 K (4), 320 K (5), (**c**). Changes in photocurrent (1) and photovoltage (2) in a magnetic field as a function of temperature (**d**).

As the concentration increases, the functional dependence of the current on the illumination intensity of the MnZn_0.85_Cr_0.15_Sb changes qualitatively (Figure 12a). The photocurrent increases sharply when the power of incident illumination per sample is increased up to 0.06 mW. A further increase in the illuminance changes the current slightly. The photovoltage increases sharply up to 0.15 mW and increases smoothly under visible light (Figure 12b). This is related to the localization of excited electrons with energies greater than 1 eV on defects.

The photovoltage dependence of the photocurrent is nonlinear in the IR region in the magnetically ordered state and becomes linear above 320 K (Figure 12c). Above this temperature, the photocurrent and photovoltage increase in a magnetic field (Figure 12d). In the magnetically ordered state, the photocurrent decreases and the photovoltage increases in a magnetic field.

**Figure 13:**
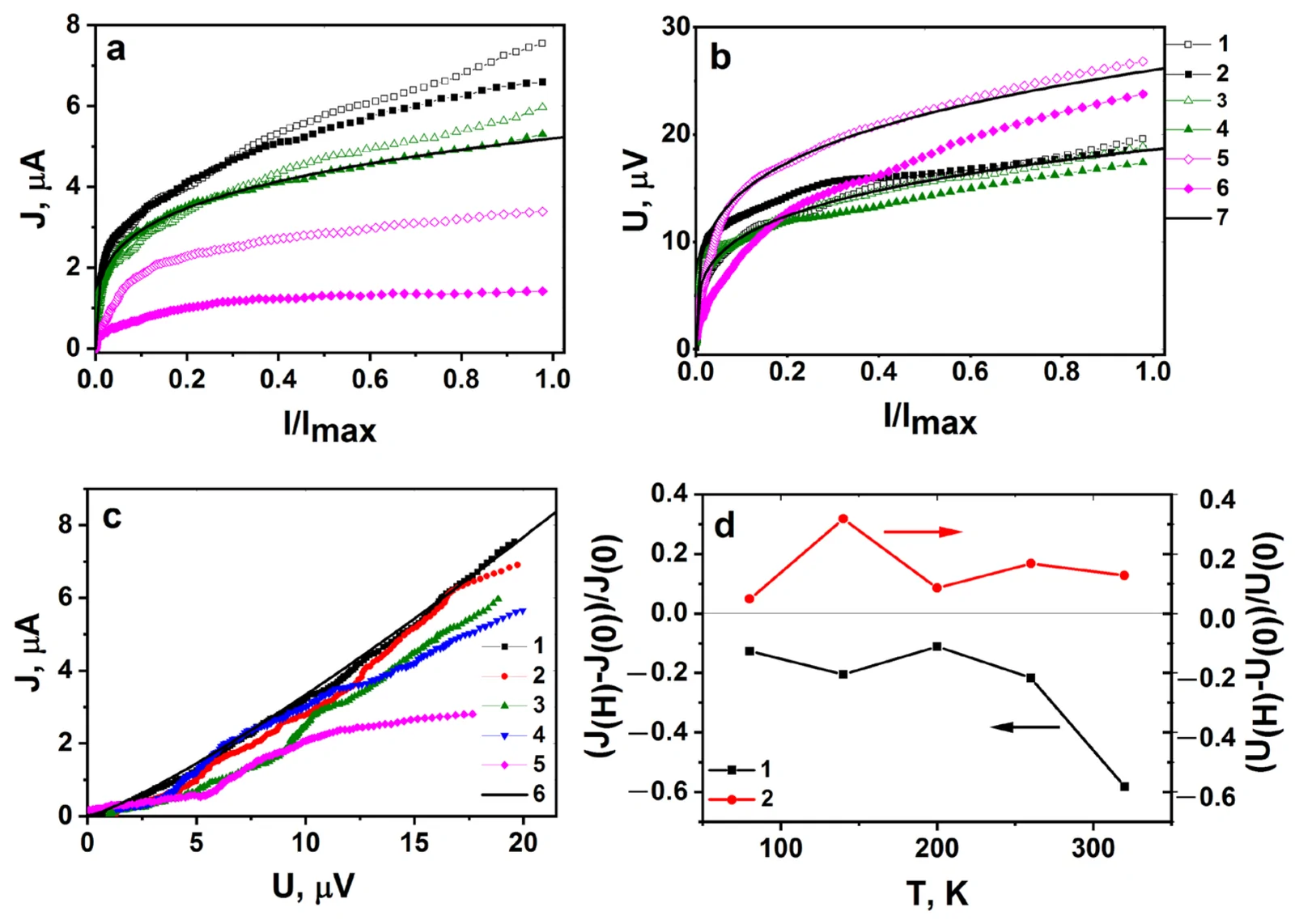
The photocurrent (**a**) and photovoltage f (**b**) on the normalized power of incident illumination in the MnZn_0.8_Cr0.2Sb sample illuminated by an incandescent lamp without a field (1,3,5) and in a magnetic field H = 1.2 kOe (2,4,6) at T = 80 K (1, 2), 200 K (3,4), 320 K (5,6), fitting functions J = A(I/Im)^n^ (7). Dependence of photocurrent on photovoltage and fitting functions J = A U^1.2^ (6) (**c**) without a magnetic field at T = 80 K (1), 140 K (2), 200 K (3), 260 K (4), 320 K (5). Changes in photocurrent (1) and photovoltage (2) in a magnetic field as a function of temperature (**d**).

In the MnZn_0.8_Cr_0.2_Sb sample, the dependences of the photocurrent and voltage on the normalized power of incident illumination (Figure 13a) are approximated by the power function I = A(I/I_m_)^n^, where the exponent n is 0.25 ± 0.0028–0.29 ± 0.003. This relation correlates with Wien’s law, in which the frequency corresponding to the maximum spectral density of radiant energy is proportional to ν_m_ ~ *I*^1/4^. The photocurrent and photovoltage are proportional to the energy of electromagnetic radiation quanta and the spectral density of the radiation is described by the Planck function. The photocurrent is proportional to the photovoltage (Figure 13c). In a magnetic field directed along the current, the photocurrent decreases and has a minimum near the magnetic phase transition. The photovoltage increases in a magnetic field. This effect is caused by the electron localization and reduced carrier mobility in a magnetic field.

## 4. Model

A qualitative explanation of the experimental results is based on theoretical calculations of the electronic structure for MnZnSb. According to first-principles calculations, the electron density of spin-down and spin-up states is continuous in MnZnSb, and Weyl nodes are located below and above the Fermi surface at some points in the Brillouin zone. The presence of Weyl nodes leads to a change in the electron density of states. The inclusion of spin–orbit coupling (SOC) leads to the formation of four pairs of Weyl nodes in the Brillouin zone, which act as the source and sink of significant Berry flux [26].

The substitution of zinc with chromium at position (II) in the inset of Figure 1 leads to the formation of a localized moment on the chromium ions and to a complex magnetic structure. The substitution of manganese for zinc at this position leads to the formation of a skyrmion lattice in Mn_2−x_Zn_x_Sb [42]. The interaction of electron spins with a localized magnetic moment leads to a shift of the electron bands in energy and momentum. The Weyl node shift upon symmetry breaking due to magnetic doping in Weyl semimetals was theoretically investigated [50]. A shift of Weyl nodes to different energy levels has been observed, leading to a transition of the nodal-point semimetal into a state with electron and hole Fermi surfaces. The topological properties of a Weyl semimetal—namely, chiral edge states and the corresponding Hall conductivity—remain under moderate symmetry breaking. The presence of electron and hole Fermi surfaces can be interpreted as the existence of electrons and holes and the possibility of a photoelectric effect in a magnetic semimetal.

The photocurrent generation in WSMs requires symmetry breaking, tilted cones in the electron excitation spectrum, Hamiltonian terms cubic with respect to the quasi-momentum, and light propagation perpendicular to the 4th-order rotation axis. Strong electron–electron interactions can break the translational symmetry and shift the energy positions of nodes [51]. The shift of Weyl nodes forms states with the electron and hole Fermi surfaces; the topological properties of the WSM state, i.e., the chiral edge states and the corresponding Hall conductivity, are preserved at the moderate symmetry breaking [51,52]. The surface treatment of the sample, local nonstoichiometry, and potential gradients near crystallite boundaries can cause asymmetry in the observed surface energy levels.

Thus, the asymmetry is an inherent feature of real materials, in which structural and orbital effects disrupt the high degree of symmetry present in simple theoretical models.

**Figure 14:**
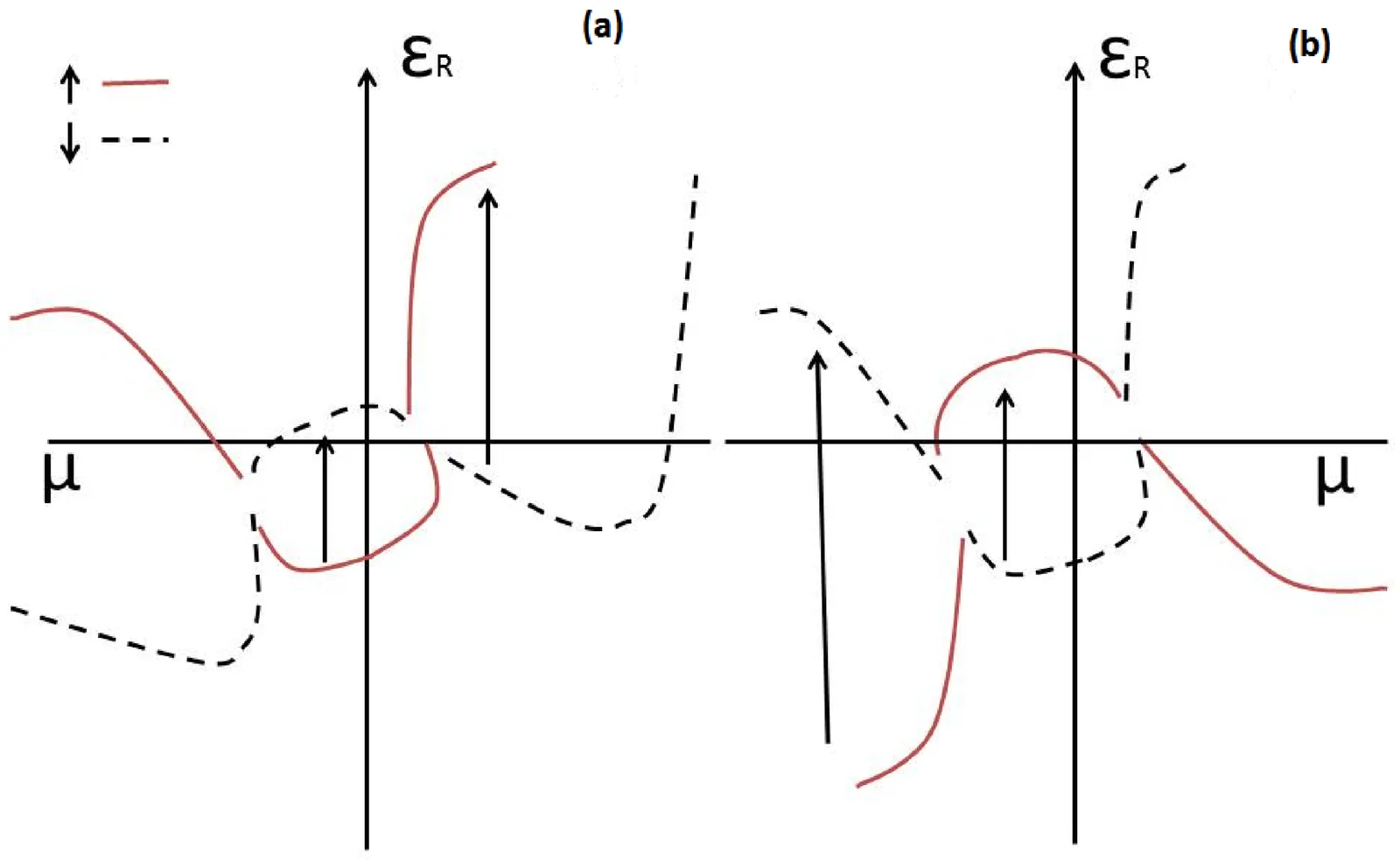
Model of the electron spectrum of MnZn_1−x_Cr_x_Sb in the center of the Brillouin zone in different planes of the tetragonal lattice with spin polarization (spin up—red line, spin down—dotted line). Weyl nodes are located below the chemical potential in the valence band (**a**) and Weyl nodes are located above the chemical potential (**b**).

Asymmetry of Weyl nodes relative to the Fermi level can be caused by asymmetry of contacts on the sample, impurities, and defects. During the thermobaric treatment of the powders at a pressure of ~5.0 GPa and a temperature of ~623 K in air, defects and charged vacancies can form nonuniformly distributed over the sample. The electric field is induced by the concentration gradient of charged defects that lead to a shift of Weyl nodes relative to the chemical potential (Figure 14). For each monopole in the Brillouin zone, there is an antimonopole, i.e., they are created and annihilated in pairs. At the intersection of the upper and lower Weyl cones, the degeneracy is lifted by spin–orbit couplings of about 20–40 meV. A 10% shift of Weyl nodes is equivalent to the appearance of an internal electric field and effective electric polarization, as in the MnPSe_3_ compound [53,54]. The lower and upper subbands with different spin directions are formed and electrons pass between them under the action of IR radiation. According to the LDA calculation, in the kx–ky plane (kz = 0), Weyl nodes are located below the chemical potential in the valence band with a width of 0.4 eV (Figure 14a), while in the kz–ky plane (kx = 0), they are above the chemical potential with a band width of 1.5 eV (Figure 14b). The electron excitation spectrum with different spin directions and electron transitions between Weyl subbands is shown in Figure 14.

The photoelectric effect depends on the concentration of chromium substitution as a result of the Fermi level shift. In the IR region with an incident power of illumination per sample up to 0.06 mW, the photocurrent sensitivity increases from 1.5 mA∙W^−1^ at x = 0.1 to 20 mA∙W^−1^ in the MnZn_0.8_Cr_0.2_Sb sample. In the TaAs material, the photovoltaic effect in the photon energy range of 25–350 meV is significantly enhanced at longer wavelengths and the photocurrent is ~2.5 μA at an incident light power of 66 mW [55]. The giant photocurrent was found experimentally in the type-II WSMs (TaIrTe_4_) at a wavelength of 1.5 μm with a sensitivity of 22.4 mA W^−1^. At a wavelength of 4 µm, it differs strikingly in the low-power region, where it is more than three orders of magnitude greater, reaching its maximum value of 130.2 mA∙W^−1^ [11].

Weyl semimetals are promising for detecting infrared (IR) and terahertz radiation. Topological semimetal detectors are characterized by ultrafast operation speed, a very broad-spectrum range, and room-temperature operation, multi optical parameter sensitivity [56].

The metal photodetector ZrGeSe with non-mirrorsymmetric properties facilitates highly effective photoelectric conversion, with a peak responsivity greater than 0.11 A/W, a response time of 8.3 ms, for 0.26 THz [57], the type-II Dirac semimetal CoTe_2_ demonstrates notable photoresponsivity exceeding 0.1 A/W, a swift response time near 710 ns, PdTe_2_ with a responsivity of 0.2 A/W for 0.3 THz [58]. A photodetector based on the layered Type-II Weyl semimetal in device metal–MoTe_2_–metal exhibits a responsivity of 0.40 mA/W and a 43 μs response time for a 532 nm laser [59]. WTe_2_ reveals an anomalous photocurrent of 40 µA and exhibits light polarization dependence with responsivity 250 A/W for a 3.8 µm laser at 77 K [60].

Photodetectors based on the Weyl semimetal of tantalum phosphide (TaP) have an ultra-wideband spectral response from the visible range to infrared and terahertz radiation with a photosensitivity of tens to hundreds of microamperes per watt (μA/W) depending on the wavelength [61].

## 5. Conclusions

The photovoltaic effect that can be controlled by a magnetic field was found in the MnZn_1−x_Cr_x_Sb Weyl semimetals. Extreme changes in the photocurrent and photovoltage upon variation in both temperature and magnetic field were observed at low temperatures. Localization of photoelectrons in a magnetic field leads to a decrease in the current and an increase in the photovoltage. A correlation was established between the temperature behavior of the photovoltage and photocurrent and the temperature behavior of the magnetization. Near the Curie temperature, the photovoltage and photocurrent decrease sharply and, above T_C_, they vary smoothly. With an increase in the concentration of chromium, the elementary tetrahedral cell stretches along the tetrahedron axis by δ (c/a) = 0.7%, the Curie temperature increases by 3%, the generation of photocurrent increases by 50% and photocurrent sensitivity increases from 1.5 mA/W to 20 mA/W.

The nonlinear dependences of the photocurrent and photovoltage on the power of incident illumination over a wide frequency range were established. The maximum sensitivity of the photocurrent in the mid-IR range increases with the chromium concentration in the MnZn_1−x_Cr_x_Sb semimetals. The photocurrent and photovoltage depend on the spectral range and, under the same illuminance from a laser, led lamp, and incandescent lamp, the maximum photoelectric effect is achieved with the latter. The chromium concentration at which the resistance increases under illumination was determined. The change in the photoresistance in a magnetic field was attributed to the localization of electrons in the vicinity of a Weyl node, which has the nodal annular dispersion. The photovoltage and photocurrent were explained by the shift of Weyl nodes relative to the chemical potential and the transition of electrons between the upper and lower spin-polarized subbands as a result of the spin–orbit coupling.

## Figures and Tables

**Table 1:** Semi-quantitative analysis of these compounds MnZn_x−1_Cr_x_Sb.

Element		X = 0.05		X = 0.1		X = 0.2	
	AN	Norm. C (wt.%)	Atom. C (at.%)	Norm. C (wt.%)	Atom. C (at.%)	Norm. C (wt.%)	Atom. C (at.%)
Sb	51	51.63	34.41	55.07	37.91	52.68	34.63
Zn	30	25.04	31.07	26.88	34.46	16.18	19.80
Mn	25	22.53	33.27	16.88	25.75	28.59	41.64
Cr	24	0.80	1.25	1.17	1.88	2.55	3.93
Total	100	100	100	100	100	100	100

## Data Availability

The original contributions presented in this study are included in the article. Further inquiries can be directed to the corresponding author.

## Abbreviations

The following abbreviations are used in this manuscript:

WSMs	Weyl semimetals
MnZn_1−x_Cr_x_Sb	Manganese-zinc- chromium antimonide
MnGeO_3_	Manganese carbonate
PrGeAl	Praseodymium-germanium-aluminum
TaAs	Tantalum arsenate
MnPSe_3_	Manganese Phosphorus Triselenide
BPV	Bulk photovoltaic effect
IR	Infrared spectroscopy
TSMs	Topological semimetals
PBE	Perdew–Burke–Ernzerhof functions
XRD	X-ray diffraction analysis
Cu_2_Sb	Cuprostibite
PbFCl	Matlockite
LA	Low-angle
HA	High-angle
LED	Light-emitting diode lamp
MnZnSb	Manganese-zinc antimonide
SOC	Spin–orbit coupling
LDA	Linear discriminant analysis
TaIrTe_4_	Tantalum iridium tetra telluride
